# The Novel Methods for Analysis of Exosomes Released from Endothelial Cells and Endothelial Progenitor Cells

**DOI:** 10.1155/2016/2639728

**Published:** 2016-03-28

**Authors:** Jinju Wang, Runmin Guo, Yi Yang, Bradley Jacobs, Suhong Chen, Ifeanyi Iwuchukwu, Kenneth J. Gaines, Yanfang Chen, Richard Simman, Guiyuan Lv, Keng Wu, Ji C. Bihl

**Affiliations:** ^1^Department of Pharmacology and Toxicology, Boonshoft School of Medicine, Wright State University, Dayton, OH 45435, USA; ^2^Department of Cardiology, The Affiliated Hospital of Guangdong Medical University, Zhanjiang, Guangdong 524001, China; ^3^College of Health Science, Wuhan Sports University, Wuhan, Hubei 430079, China; ^4^Departments of Neurology and Internal Medicine, Boonshoft School of Medicine, Wright State University, Dayton, OH 45435, USA; ^5^Zhejiang Chinese Medical University, Hangzhou, Zhejiang 310053, China; ^6^Department of Neurology, Ochsner Medical Center, Jefferson, LA 70121, USA

## Abstract

Exosomes (EXs) are cell-derived vesicles that mediate cell-cell communication and could serve as biomarkers. Here we described novel methods for purification and phenotyping of EXs released from endothelial cells (ECs) and endothelial progenitor cells (EPCs) by combining microbeads and fluorescence quantum dots (Q-dots®) techniques. EXs from the culture medium of ECs and EPCs were isolated and detected with cell-specific antibody conjugated microbeads and second antibody conjugated Q-dots by using nanoparticle tracking analysis (NTA) system. The sensitivities of the cell origin markers for ECs (CD105, CD144) and EPCs (CD34, KDR) were evaluated. The sensitivity and specificity were determined by using positive and negative markers for EXs (CD63), platelets (CD41), erythrocytes (CD235a), and microvesicles (Annexin V). Moreover, the methods were further validated in particle-free plasma and patient samples. Results showed that anti-CD105/anti-CD144 and anti-CD34/anti-KDR had the highest sensitivity and specificity for isolating and detecting EC-EXs and EPC-EXs, respectively. The methods had the overall recovery rate of over 70% and were able to detect the dynamical changes of circulating EC-EXs and EPC-EXs in acute ischemic stroke. In conclusion, we have developed sensitive and specific microbeads/Q-dots fluorescence NTA methods for EC-EX and EPC-EX isolation and detection, which will facilitate the functional study and biomarker discovery.

## 1. Introduction

Exosomes (EXs) are nanoscale extracellular vesicles that are derived from the multivesicular endosomal cell compartment [1–3]. Upon release, EXs may either circulate in the extracellular space adjacent to the site of release or enter into biological fluids (e.g., plasma, urine, and cerebrospinal fluid). Recent studies have indicated that EXs carry the genetic and proteomic contents of their parent cells [4, 5]. Moreover, increasing evidence has demonstrated that EXs are important mediators of cell-to-cell communication and play crucial roles in both physiological and pathophysiological processes. They have been shown to be involved in inflammation, tumorigenesis, cardiovascular diseases, and so forth [6–10]. Thus, a better understanding of the phenotype of EXs in biofluids is required. However, limited studies have shown the isolation and detection protocols of specific EXs from biofluids. Although the conventional EX isolation techniques, such as ultracentrifugation and density-gradient separation, can achieve collection of EXs, these techniques cannot separate particular phenotype of EXs from the EX compound due to their similar size and buoyant density [11].

Microbeads are superparamagnetic particles that are conjugated to highly specific antibodies against a particular antigen on the cell surface. They are often used to isolate and enrich specific cell subpopulations via their coated antibodies [12]. Since EXs carry the antigens of their parent cells, it is logical to assume that specific antigen-conjugated microbeads could be used for EX isolation, purification, and enrichment. Nanoparticle tracking analysis (NTA) is a new technology that can detect vesicles as small as 30 nm in diameter [13, 14] and count specific subgroups of EXs using antibodies conjugated to fluorescent probes called quantum dots (Q-dots) [6, 15]. The application of Q-dots and fluorescence NTA to phenotype specific circulating extracellular vesicles, syncytiotrophoblast-derived microvesicles, and epithelial tumor cell-derived EXs has been demonstrated [15, 16]. Based on these observations, we hypothesized that cell-specific antibody conjugated microbeads combined with fluorescence Q-dots are able to isolate and phenotype EXs from biofluids, especially for those that expose more than one surface antigen of their parent cells.

In this study, for developing the methods, we used one of the specific surface antigens of endothelial cells (ECs) or endothelial progenitor cells (EPCs) to capture the EXs released from cultured ECs or EPCs, and then we probed with other EC or EPC specific surface antigens to phenotype the captured EXs. To the best of our knowledge, this is the first work describing the combination of microbeads, fluorescence Q-dots, and NTA to detect specific EXs. Moreover, we could accurately numerate EC-EXs and EPC-EXs from human plasma by using the methods.

## 2. Materials and Methods

### 2.1. Preparation of EXs from Cell Culture Medium

Human brain microvascular ECs were purchased from Cell Systems (Kirkland, WA) and cultured in CSC complete medium containing 10% serum, 2% human recombinant growth factors, and 0.2% antibiotic solution under standard cell culture conditions (5% CO_2_, 37°C). Cell medium was changed twice a week. Passages 4 to 13 of ECs were used for experiments in this study. EPCs were purchased from Amsbio. For EC-EX preparation, ECs were challenged with CSC medium (Cell Systems) supplemented with 2% human recombinant growth factors and 0.2% antibiotic solution for 24 hr. For EPC-EX preparation, EPCs were challenged with EPC basal medium (Amsbio) and 0.2% antibiotic solution for 24 hr. Then, the cell medium was collected and centrifuged at 300 g for 15 min, followed by centrifugation at 2000 g for 30 min to remove cells and cell debris. The cell-free culture medium was centrifuged at 20,000 g for 70 min and then ultracentrifuged at 170,000 g for 6 hr to pellet EXs. The pelleted EXs were resuspended with 20 nm filtered (Whatman, Pittsburgh, PA) phosphate-buffered saline (PBS) and aliquoted for NTA.

### 2.2. Isolation of EC-EXs and EPC-EXs from EC and EPC Culture Medium by Using Anti-CD105- or Anti-CD34-Conjugated Microbeads

According to the manufacturer's instruction with modifications, the pelleted EXs were incubated with 10 *μ*L of Biotin-conjugated anti-CD105 (specific for ECs), anti-CD41 (specific for platelets), anti-CD235a (specific for erythrocytes), or anti-CD34 (specific for EPCs) antibody (Miltenyi Biotec) in a 100 *μ*L reaction volume for 2 hr, followed by adding 10 *μ*L of anti-Biotin microbeads (Miltenyi Biotec), respectively. Then, a magnet module was applied to separate microbeads-labeled EXs from the total EX suspension. After an overnight magnet separation, the fluid was gently removed from the magnet and was considered as wastes. The microbeads bound EXs were resuspended with 100 *μ*L filtered PBS and added with 10 *μ*L of multisort release reagent (Miltenyi Biotec) for 10 min to cleave off the microbeads from EXs by following the manufacturer's instruction. Then, all samples were added with 150 *μ*L filtered PBS to bring the final volume to 250 *μ*L and were placed at a magnet (DynaMag-2; Life technology) to get rid of the released microbeads overnight. On the following day, the fluid was collected and considered as CD105^+^, CD41^+^, CD235a^+^, or CD34^+^ EXs. All isolated EXs were enumerated by using the NTA NS300 system (Malvern Instruments). The purification efficiency was calculated as the number of microbead positive EXs divided by the total number of EXs.

### 2.3. Immunofluorescence Labeling of CD105^+^ EXs and CD34^+^ EXs with Q-Dots

The isolated CD105^+^ or CD34^+^ EXs were then incubated with other sets of primary antibodies, goat-against CD144 (specific for ECs), goat-against KDR (specific for EPCs), goat-against Annexin V (specific for MVs), or goat-against CD63 (specific for EXs) (1 : 200 dilution; Santa Cruz Biotechnology), for 2 hr, followed by incubation with rabbit anti-goat IgG conjugated with Q-dot 655 (1 : 350 dilution; Life Technologies) for 90 min at RT. Filtered PBS was added to EX suspension to give a final volume of 700 *μ*L. All samples were analyzed by the NTA NS300 system (Malvern Instruments, United Kingdom).

### 2.4. Nanoparticle Tracking Analysis

The NanoSight NS300 with a 405 nm laser instrument (Malvern Instruments, United Kingdom) was used to detect EXs without label or labeled with stable fluorophores. The NanoSight polystyrene latex calibration beads, 100 nm and 200 nm, were applied to check the instrument performance. In this study, diluted suspensions containing EXs were loaded into the sample chamber, and the camera level was maintained at 10 for light scatter mode and at 16 for fluorescence scatter mode between samples. Light scatter mode of NTA used the camera filter 1, and fluorescence mode used the camera filter 2 with the long-pass 430 nm filter in place. Three videos of typically 30 seconds duration were taken, with a frame rate of 30 frames per second. Data was analyzed by NTA 3.0 software (Malvern Instruments) which was optimized to first identify and then track each particle on a frame-by-frame basis.

### 2.5. Calculation of the Detection and Overall Efficiencies of Double Labeled EXs

The detection efficiency of the CD105^+^Q-dots^+^ EXs and CD34^+^Q-dots^+^ EXs was calculated as follows: (CD105^+^Q-dots^+^) EXs% = (the number of CD105^+^Q-dots^+^ EXs)/(the total number of CD105^+^ EXs); (CD34^+^Q-dots^+^) EXs% = (the number of CD34^+^Q-dots^+^ EXs)/(the total number of CD34^+^ EXs). The overall efficiency of measuring the CD105^+^Q-dots^+^ EXs or CD34^+^Q-dots^+^ EXs was calculated as follows: (CD105^+^) EXs% = (the number of CD105^+^Q-dots^+^ EXs)/(the total number of EXs); (CD34^+^) EXs% = (the number of CD34^+^Q-dots^+^ EXs)/(the total number of EXs).

### 2.6. Protein and Western Blot Assays

Proteins from EC-EXs and EPC-EXs were isolated with lysis buffer (Thermo Scientific, FL) containing protease inhibitor. Protein concentration assay was conducted using a Bradford assay kit (Bio-Rad Laboratories). The linear range of the assay for BSA is from 0.2 to 0.9 mg/mL. Plates were read at 595 nm using a spectrofluorometer (BioTek Instruments). For western blot analysis, the proteins were subjected to electrophoresis and transferred onto PVDF membranes. The membranes were blocked by incubating with 5% dry milk for 1 hr, followed by incubation with primary antibodies overnight at 4°C. The primary antibodies used were anti-CD63 (1 : 400; BD Biosciences), anti-CD105 (1 : 500; Santa Cruz), anti-CD34 (1 : 500, Santa Cruz), and *β*-actin (1 : 4000, Sigma). After being washed thoroughly, membranes were incubated with horseradish peroxidase-conjugated IgG (1 : 40000; Jackson ImmunoResearch Labs) for 1 hr at room temperature. Blots were then developed with enhanced chemiluminescence developing solutions.

### 2.7. TEM of EC-EXs and EPC-EXs

The EXs collected from EC and EPC conditioned medium were fixed with 2% glutaraldehyde and postfixed with 1% osmium (all were purchased from Electron Microscopy Science, Hatfield, PA), and then they were embedded with Spurr resin (Sigma, St. Louis, MO) and baked at 60°C according to the manufacturer's instruction and our previous study [17]. Ultrathin sections (60–80 nm) were prepared with MT7000, mounted on 300-mesh copper grids, and stained with uranyl acetate and lead citrate. All samples were examined with an EM 208 (Philips) transmission electron microscope at an accelerating voltage of 70 KV.

### 2.8. Recovery of EC-EXs and EPC-EXs from Particle-Free Plasma

The human plasma was diluted 5x with filtered PBS and centrifuged at 200 g for 20 min. The supernatant was centrifuged at 20,000 g for 70 min, followed by ultracentrifugation at 170,000 g for 6 hr. The supernatant after ultracentrifugation was analyzed by NTA and considered particle-free plasma. The known amounts (6 × 10^8^ particles) of EC-EXs or EPC-EXs that were isolated from ECs and EPCs were added into 1 mL of prepared particle-free plasma. The EC-EXs/particle-free plasma and EPC-EXs/particle-free plasma mixture were centrifuged at 20,000 g for 70 min at 4°C and then ultracentrifuged at 170,000 g for 6 hr to pellet recovered EC-EXs and EPC-EXs. The recovered EC-EXs and EPC-EXs were resuspended with filtered PBS and then isolated by anti-CD105-, anti-CD34-, anti-CD41-, or anti-CD235-conjugated microbeads and analyzed by NTA. Likewise, the recovered microbeads positive EXs were probed with anti-CD144-, anti-Annexin V-, anti-KDR-, or anti-CD63-conjugated Q-dots and then subsequently analyzed by fluorescence NTA. The recovery rate was calculated as the number of microbeads positive EXs divided by the total number of EXs. Similarly, the detection and overall efficiency of the recovered CD105^+^Q-dots^+^ EXs and CD34^+^Q-dots^+^ EXs were calculated as described above.

### 2.9. Study Subjects

This study recruited 16 ischemic stroke patients from the Department of Neurology at the Ochsner Medical Center. Peripheral blood (3 mL) was collected from ischemic stroke patients on admission day (day 1) after stroke occurs. 16 patients were divided into two groups: one group for cEC-EXs analysis (*n* = 8); another group was used to detect cEPC-EXs (*n* = 8). Exclusion criteria of subjects for this study included any of the following situations: (1) infectious disease in a previous month; (2) histories of autoimmune disorder, peripheral vascular disease, or stroke; (3) transient ischemic attack, cerebral infarction, and cerebral hemorrhage; (4) liver failure and acute or chronic kidney disease; (5) recent myocardial disease in the last 3 months; (6) medications for lipid control, inflammation suppression, or immunosuppression; and (7) history of cancer. All experiment protocols were approved by the Department of Neurology at the Ochsner Medical Center and IRB committee at Wright State University. Written informed consent was obtained from each participant prior to enrollment in the study.

### 2.10. Preparation and Analyses of Circulating EXs from Human Plasma

Whole blood samples (3 mL) were drawn from ischemic stroke patients at admission day (day 1) and days 3 and 5 after admission using tubes containing 3.13% sodium citrate. The whole blood samples were diluted 3x with filtered PBS and centrifuged at 400 g for 35 min at 4°C; the uppermost layer was collected as plasma. 1 mL of plasma was centrifuged at 2000 g for 20 min to remove platelet. The supernatant was collected and centrifuged at 20,000 g for 70 min, and then the supernatant was ultracentrifuged at 170,000 g for 6 hr to pellet circulating EXs (cEXs). The pelleted cEXs were resuspended with filtered PBS and then isolated by anti-CD105- or anti-CD34-conjugated microbeads and analyzed by NTA. Then, the microbeads captured cEXs were probed with anti-CD144-, anti-Annexin V-, anti-KDR-, or anti-CD63-conjugated Q-dots and then subsequently analyzed by fluorescence NTA. The proportion of cEXs using fluorescence NTA was calculated as (CD105^+^Q-dots^+^) cEXs% = CD105^+^Q-dots^+^ cEXs/total cEXs; (CD34^+^Q-dots^+^) cEXs% = CD34^+^Q-dots^+^ cEXs/total cEXs. The absolute number of cEC-EXs and cEPC-EXs were the absolute counts of CD105^+^CD144^+^ cEXs or CD34^+^KDR^+^ cEXs per mL human plasma.

### 2.11. Statistical Analysis

Experimental data were expressed as the mean ± SEM and were analyzed using one- or two-way analysis of variance (ANOVA). The correlation of EX numbers with their protein concentrations was analyzed using Spearman's rank correlation test (SPSS version 17.0; SPSS, Chicago, IL). Values of *P* < 0.05 were considered to be of statistical significance.

## 3. Results and Discussion

### 3.1. EXs Isolated from EC and EPC Culture Medium Were Characterized by NTA, TEM, and Western Blot Analyses

**Figure 1:**
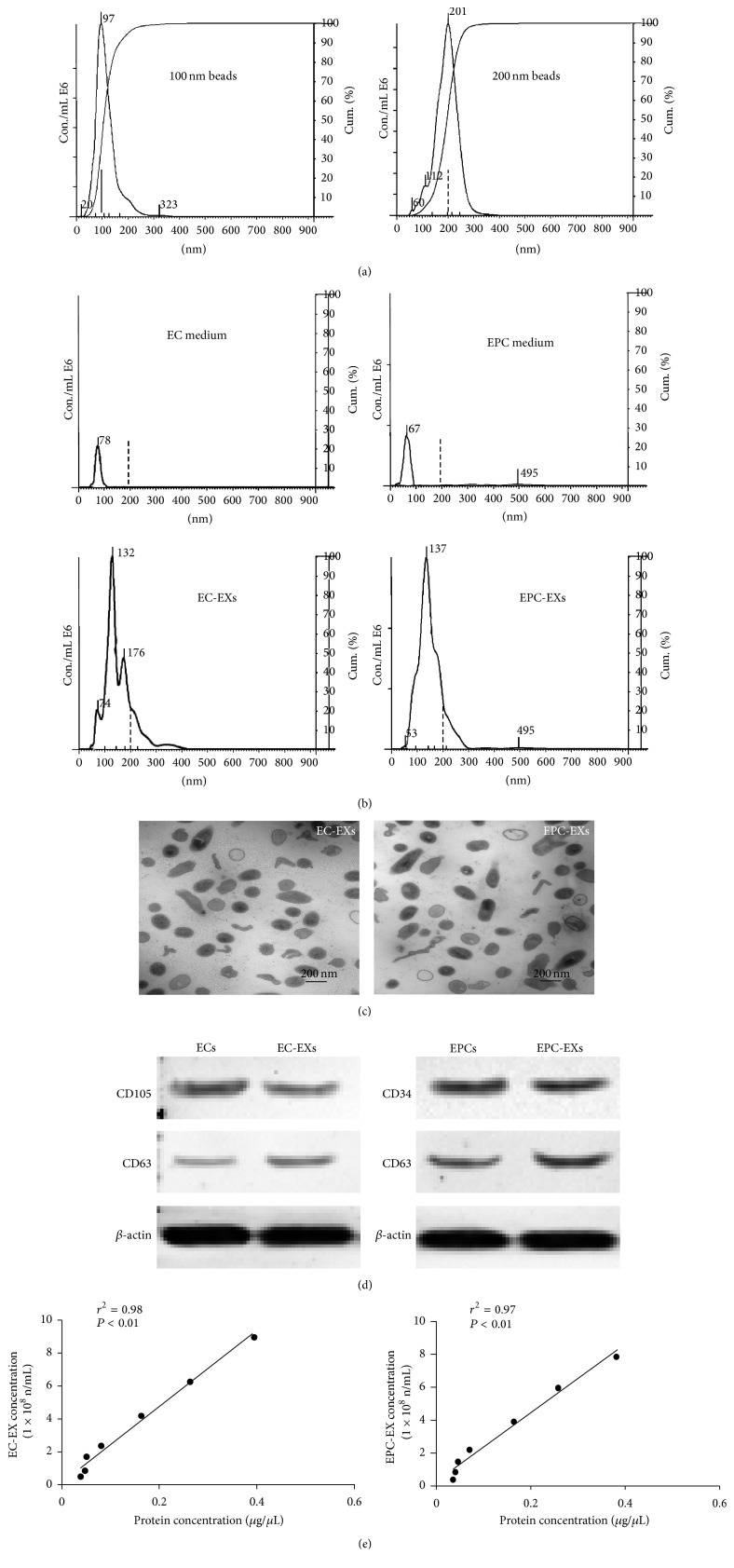
Characterization of EC-EXs and EPC-EXs. (a) Representative NTA plot showing the size distribution of 100 nm and 200 nm polystyrene beads. (b) Representative NTA plots show size/concentration distribution of particles in the EX-depleted EC and EPC medium and of EC-EXs and EPC-EXs. Black solid line: 100 nm landmark; black dash line: 200 nm landmark. (c) TEM micrographs of EXs. (d) Representative western blot bands showing the expression of CD63, CD105, and CD34 in EXs and their corresponding parent cells, ECs and EPCs. (e) The correlation between EXs and their protein concentration.

The Nanosight NS300 system was calibrated with 100 nm and 200 nm polystyrene beads (Figure 1(a)) prior to detecting experimental samples. As shown in Figure 1(b), there were only few particles left in the EX-depleted EC medium and EPC medium. The average size of EC-EXs was 158 ± 55 nm and of EPC-EXs was 154 ± 59 nm, which were consistent with previous observations [18, 19]. Meanwhile, our TEM results were in accordance with the NTA data showing that the EC-EXs and EPC-EXs were made up of small particles less than 200 nm in diameter (Figure 1(c)). In addition, our western blot results showed that the EC-EXs and EPC-EXs expressed CD63 which is one of the protein families most commonly associated with EXs and is generally used as EX markers [20, 21], further confirming that the microbeads-isolated particles were EXs. Meanwhile, it is not surprising to find that EC-EXs express their parent cell marker CD105 and that EPC-EXs express EPC specific marker CD34 (Figure 1(d)). In future studies, other EC and EPC specific markers such as CD31 and CD133 could be tested in EC-EXs and EPC-EXs released from ECs and EPCs under different conditions, respectively. As many previous studies used the protein concentration of EXs, not the particle number, as a quantification parameter for their functional analyses [22–24], we analyzed the correlation between protein concentration and the particle number of EC-EXs or EPC-EXs. The correlation coefficient plots demonstrated that the concentration of EC-EXs and EPC-EXs detected by NTA highly positively correlated with their respective protein concentration measured by Bio-Rad protein assay (Figure 1(e)). This correlation could be applied to deduce protein concentration from the particle numbers for* in vivo* experiments in the future.

### 3.2. EC-EXs and EPC-EXs Were Isolated by Combining with Anti-CD105- or Anti-CD34-Conjugated Microbeads and Anti-CD144- or Anti-KDR-Conjugated Q-Dots

**Figure 2:**
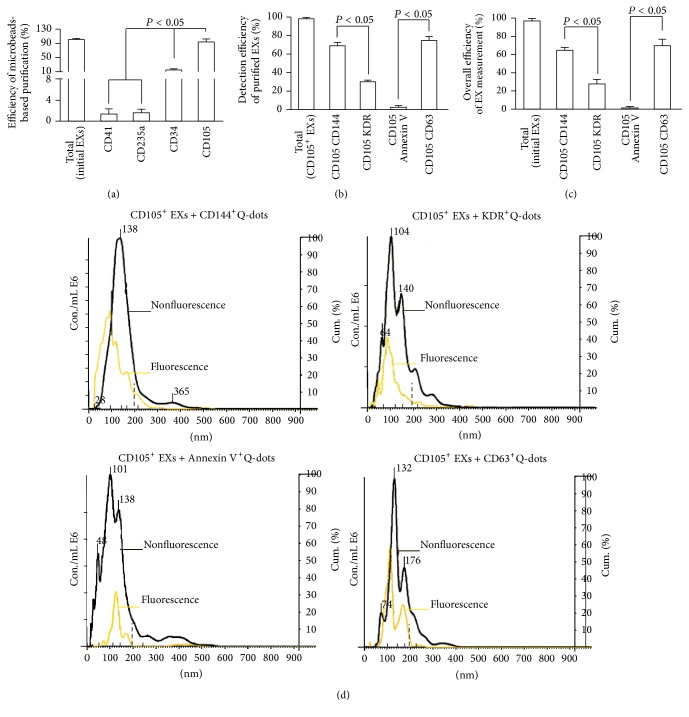
The efficiencies and specificities of the methods by combining microbeads with NTA for purifying and detecting EC-EXs. (a) The purification efficiency and specificity of EC-EXs in the total EC-EXs, which were collected from EC culture medium by ultracentrifuge and isolated by various microbeads-conjugated antibodies against EC specific markers (CD105, as well as negative controls, CD34, CD41, and CD235a). (b) The detection efficiency and specificity of EC-EXs in the total CD105^+^ EXs that were labeled with CD144-, or KDR-, or Annexin V-, or CD63-conjugated Q-dots upon detection by fluorescence NTA. (c) The overall efficiency for measuring the CD105^+^ EXs colabeled with CD144-, or KDR-, or Annexin V-, or CD63-conjugated Q-dots in the total EC-EXs. (d) Representative plots showing the size/concentration distribution of the CD105^+^ beads isolated EXs under fluorescence/nonfluorescence modes. Black curve: CD105^+^ EXs measured under light scatter (nonfluorescence) mode. Yellow curve: CD105^+^Q-dots^+^ EXs measured under fluorescence mode. Black dash line: 200 nm landmark. *N* = 4/group.

**Figure 3:**
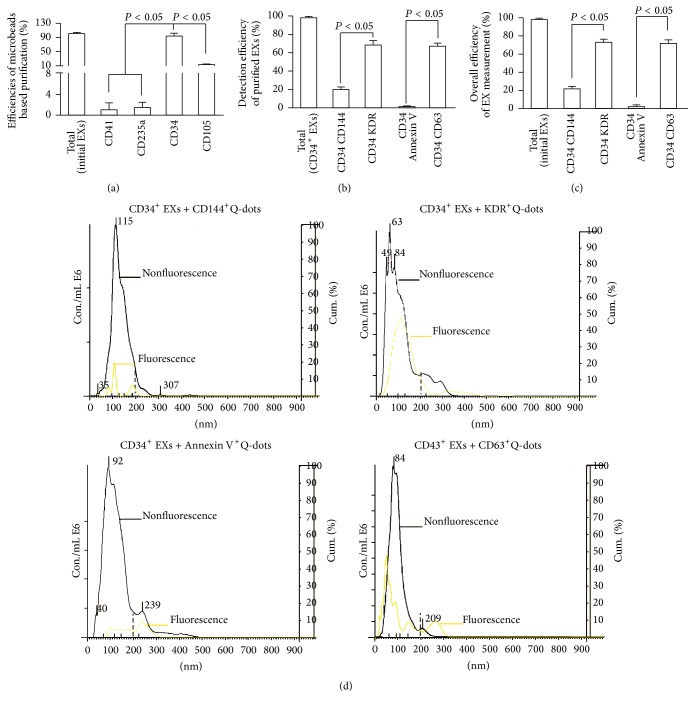
The efficiencies and specificities of the methods by combining microbeads with NTA for purifying and detecting EPC-EXs. (a) The purification efficiency and specificity of EPC-EXs in the total EPC-EXs, which were collected from EPC culture medium by ultracentrifuge and isolated by various microbeads-conjugated antibodies against EPC specific markers (CD34, as well as negative controls, CD105, CD41, and CD235a). (b) The detection efficiency and specificity of EPC-EXs in the total CD34^+^ EXs that were labeled with CD144-, or KDR-, or Annexin V-, or CD63-conjugated Q-dots upon detection by fluorescence NTA. (c) The overall efficiency for measuring the CD34^+^ EXs colabeled with CD144-, or KDR-, or Annexin V-, or CD63-conjugated Q-dots in the total EC-EXs. (d) Representative plots showing the size/concentration distribution of the CD34^+^ beads isolated EXs under fluorescence/nonfluorescence modes. Black curve: CD34^+^ EXs measured under light scatter (nonfluorescence) mode. Yellow curve: CD34^+^Q-dots^+^ EXs measured under fluorescence mode. Black dash line: 200 nm landmark. *N* = 4/group.

Firstly, in order to develop the methods, we used EC-EXs derived from cultured ECs as the standard samples for establishing the isolation method. And we also confirmed this isolation method by using EPC-EXs released from cultured EPCs. Because a panel of markers (CD34, KDR) has been used as surrogate markers for EPCs [25, 26] and CD105 and CD144 have been identified for ECs [27], we used these antibodies to establish the method. The data showed that anti-CD105-conjugated microbeads had the highest efficiency (>94%) in purifying EC-EXs (Figure 2(a)) and anti-CD34-conjugated microbeads had the highest efficiency (>93%) in purifying EPC-EXs (Figure 3(a)), when compared to those isolated by negative controls such as anti-CD41- (specific for platelets) or anti-CD235a- (specific for erythrocytes) conjugated microbeads. These results reflect the specificity of CD105 for isolating EC-EXs and CD34 for isolating EPC-EXs, and they also further provide proof for the notion that antigen expressed on EX surface can be used for their selective isolation [28].

After incubation with Q-dots-conjugated antibodies against CD144 (EC marker), KDR (EPC marker), or CD63, the purified CD105^+^ EXs and CD34^+^ EXs were analyzed by fluorescence NTA. According to the results of NTA, the detection efficiency of CD105^+^CD144^+^ EXs was around 70% and that of CD105^+^KDR^+^ EXs was only around 30% (Figure 2(b)). Likewise, the detection efficiency of CD34^+^KDR^+^ EXs was around 68% and of CD34^+^CD144^+^ EXs was only around 20% (Figure 3(b)). As revealed by the overall efficiency of the EX measurement, more than 62% of EXs isolated from EC culture medium colabeled with CD105 and CD144 (Figure 2(c)). Similarly, the majority (72%) of EXs isolated from EPC culture medium colabeled with CD34 and KDR (Figure 3(c)). In order to further exclude the contamination in particles, we incubated the anti-CD105 or anti-CD34 captured EXs with anti-CD63-conjugated Q-dots or anti-Annexin V-conjugated Q-dots. The data showed that above 70% of captured EXs were positive for CD63, whereas a few of them expressed Annexin V, suggesting that there was a low contamination of MVs in the microbeads captured EXs. Notably, the size profile of CD105^+^ EXs (black curve) overlapped with the Q-dots labeled CD105^+^ EXs (yellow curve). Likewise, the size profile of CD34^+^ EXs (black curve) overlapped with the Q-dots labeled CD34^+^ EXs (yellow curve) (Figures 2(d) and 3(d)). These data indicate that Q-dots binding did not change the physical characteristics of EXs.

Collectively, all of these results demonstrate that the methods combining microbeads and Q-dots with fluorescence NTA are able to sensitively and specifically enumerate EXs from a particle pool.

### 3.3. EC-EXs and EPC-EXs Were Recovered by Anti-CD105- or Anti-CD34-Conjugated Microbeads Combined with Anti-CD144- or Anti-KDR-Conjugated Q-Dots from Particle-Free Plasma

**Figure 4:**
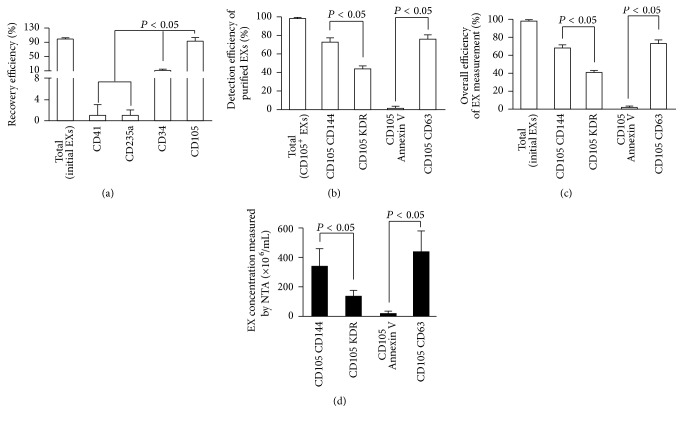
High recovery and detection efficiency of EC-EXs from particle-free plasma by using microbead purification and fluorescence NTA detection methods. (a) EC-EXs were recovered from particle-free plasma by using microbeads conjugated with various antibodies and analyzed by NTA. (b) The detection efficiency of recovered CD105^+^ EXs that were labeled with secondary antibodies (CD144, or KDR, or Annexin V, or CD63) conjugated with Q-dots and analyzed by fluorescent NTA. (c) The overall efficiency for measuring the recovered CD105^+^ EXs colabeled with CD144-, or KDR-, or Annexin V-, or CD63-conjugated Q-dots. (d) The absolute number of recovered CD105^+^ EXs that were positive for CD144, or KDR, or Annexin V, or CD63 per mL particle-free plasma. *N* = 4/group.

**Figure 5:**
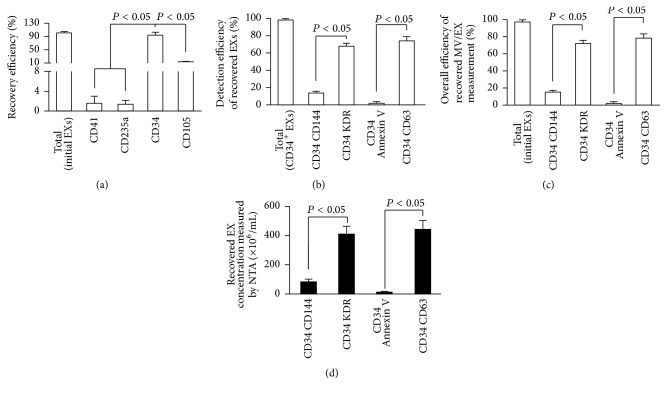
High recovery and detection efficiency of EPC-EXs from particle-free plasma by using microbead purification and fluorescence NTA detection methods. (a) EPC-EXs were recovered from particle-free plasma by using microbeads conjugated with various antibodies and analyzed by NTA. (b) The detection efficiency of recovered CD34^+^ EXs that were labeled with secondary antibodies (CD144, or KDR, or Annexin V, or CD63) conjugated with Q-dots and analyzed by fluorescent NTA. (c) The overall efficiency for measuring the recovered CD34^+^ EXs colabeled with CD144-, or KDR-, or Annexin V-, or CD63-conjugated Q-dots. (d) The absolute number of recovered CD34^+^ EXs that were positive for CD144, or KDR, or Annexin V, or CD63 per mL particle-free plasma. *N* = 4/group.

In order to test the recovery efficiency of EXs from plasma by using the above described methods, we added a known amount (6 × 10^8^ particles) of CD105^+^ EXs or CD34^+^ EXs into particle-free plasma and then assessed their respective recovery rate and detection efficiency. As described, a known amount of EXs (6 × 10^8^ particles) was added into particle-free plasma, followed by incubation with anti-CD105-, anti-CD41-, anti-CD235a-, or anti-CD34-conjugated microbeads. The data showed that almost 90% of added EC-EXs and EPC-EXs were captured by anti-CD105- (Figure 4(a)) or anti-CD34-conjugated microbeads (Figure 5(a)), whereas very few of EXs were captured by anti-CD41 or anti-CD235a (negative controls), further validating the specificity and sensitivity of the microbeads for capturing EXs. After incubation with the second antibody and Q-dots, the NTA results showed that the detection efficiency of CD105^+^CD144^+^ EXs was around 72% and of CD105^+^KDR^+^ EXs was around 42% (Figure 4(b)). And the detection efficiency of CD34^+^KDR^+^ EXs was around 69% and of CD34^+^CD144^+^ EXs was only 17% (Figure 5(b)).

As revealed by the overall efficiency of the EX measurement, more than 70% of recovered EC-EXs colabeled with CD105 and CD144 (Figure 4(c)). Similarly, the majority (65%) of recovered EPC-EXs colabeled with CD34 and KDR (Figure 5(c)). Likewise, CD63 and Annexin V were used to identify whether the particles were EXs. The data indicated that above 75% of both types of captured EXs were positive for CD63, but only few were positive for Annexin V, which was in line with the absolute numbers as shown in Figures 4(d) and 5(d). All of these data were in agreement with the results we observed in the standard EC-EX and EPC-EX samples, and they further validated the purification efficiency and specificity of the established method.

### 3.4. Isolation of cEC-EXs and cEPC-EXs in Patient Plasma of Acute Ischemic Stroke by Using Anti-CD105- or Anti-CD34-Conjugated Microbeads Combined with Anti-CD144- or Anti-KDR-Conjugated Q-Dots Methods

A previous study has shown that elevated levels of circulating CD105^+^CD144^+^ endothelial-derived extracellular microvesicles were found in plasma from patients with vascular diseases, which indicate that they could serve as a surrogate marker of endothelial function [10]. Enumeration and phenotyping of EXs are important considerations for their use in clinical studies and in the comparison of EXs from different sources. However, the nanometer size of EXs harbors the accurate counting by using flow cytometry [29, 30]. By using the methods described here, we firstly presented the proof of the concept of phenotyping EXs from plasma samples.

**Figure 6:**
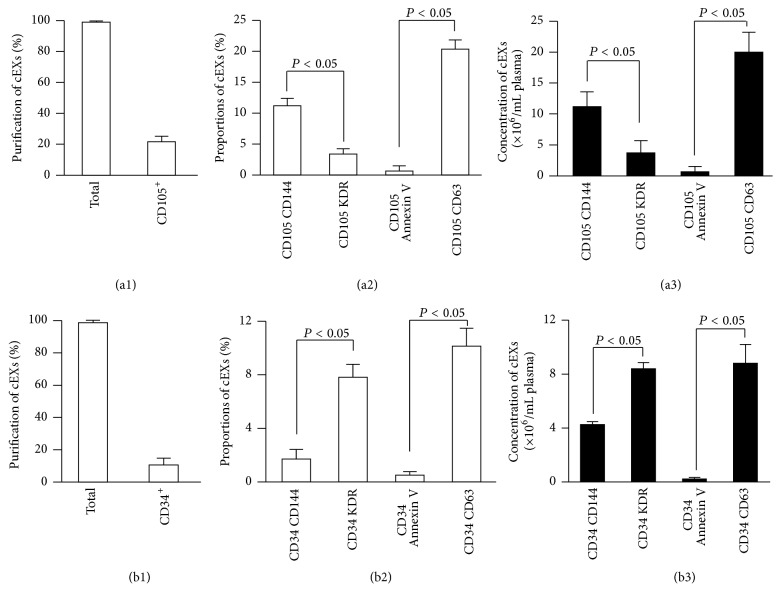
Identification of cEC-EXs and cEPC-EXs from human plasma by using anti-CD105- or anti-CD34-conjugated microbeads and Q-dots combined with fluorescence NTA. ((a1) and (b1)) The proportions of CD105^+^ cEXs and CD34^+^ cEXs in plasma that were isolated by anti-CD105- or anti-CD34-conjugated microbeads. ((a2) and (b2)) The proportion of CD105^+^ cEXs or CD34^+^ cEXs colabeled with CD144-, or KDR-, or Annexin V-, or CD63-conjugated Q-dots in total cEXs. ((a3) and (b3)) The absolute number of CD105^+^ cEXs and CD34^+^ cEXs that were labeled with CD144, or KDR, or Annexin V, or CD63 per mL day 1 ischemic stroke patient plasma. *N* = 8/group.

As shown in Figure 6, approximately 21.8% and 10.8% of cEXs were captured by anti-CD105- or anti-CD34-conjugated microbeads, respectively. With the probe of Q-dots-conjugated antibodies, we found that about 12% of total cEXs were CD105^+^CD144^+^ cEC-EXs and 7.9% of total cEXs were CD34^+^KDR^+^ cEPC-EXs. Among the collected cEXs, 20% of them coexpressed CD105 and CD63, and 10.2% of them coexpressed CD34 and CD63. There was a low percentage of cEXs expressing Annexin V, reflecting the extremely low cross-contamination of circulating MVs in the collected cEXs. There were approximately 1.15 × 10^7^ CD105^+^CD144^+^ cEC-EXs and 8.3 × 10^6^ CD34^+^KDR^+^ cEPC-EXs per mL plasma collected from day 1 after patient admission (Figures 6(a3) and 6(b3)).

### 3.5. Dynamic Changes of cEC-EXs and cEPC-EXs in Patient Plasma of Acute Ischemic Stroke

**Figure 7:**
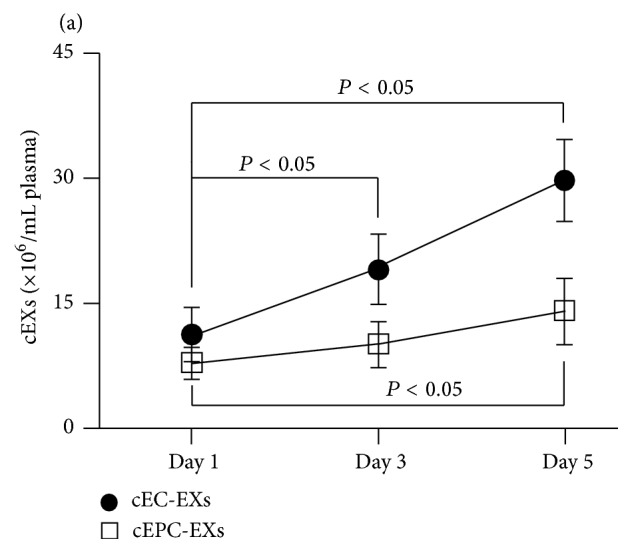
The dynamic change of cEC-EXs and cEPC-EXs in stroke patient plasma on days 1, 3, and 5 after admission. (a) The dynamic change of cEC-EXs and cEPC-EXs per mL plasma on days 1, 3, and 5 after stroke patient admission upon analysis by NTA.

Moreover, we assessed the dynamic changes of CD105^+^CD144^+^ cEC-EXs and CD34^+^KDR^+^ cEPC-EXs in patients at days 1, 3, and 5 after admission. As shown in Figure 7, we found that there were significant elevated levels of CD105^+^CD144^+^ cEC-EXs on days 3 and 5 as compared with that on day 1. The study of EC-EXs allows us to study the status of the endothelium* in vivo*, providing a novel approach that has promising potential for further understanding of stroke pathophysiology. Meanwhile, this result provides support of EC-EX as a biomarker of endothelial injury in ischemic stroke.

As analyzed by NTA, there were significant elevated levels of CD34^+^KDR^+^ cEPC-EXs on day 5 as compared with that on day 1, but there was no difference between days 1 and 3. The increase in EPC-EXs in ischemic stroke may be a result of increased number of circulating EPCs in response to ischemia. Taken together, all of these findings will be useful for developing therapy by using EXs for ischemic stroke.

## 4. Conclusion

In conclusion, in the present study, we have established sensitive and specific methods that phenotype and enumerate EXs in cell culture medium and plasma samples. Given their ease, wide applicability, and high discovery potential, we believe these methodologies could be an important addition to the technical repertoire for the qualitative and quantitative assessment of EXs in a variety of systems, ranging from cardiovascular to inflammatory diseases.
